# Highly Multiplexed RNA Aptamer Selection using a Microplate-based Microcolumn Device

**DOI:** 10.1038/srep29771

**Published:** 2016-07-19

**Authors:** Sarah J. Reinholt, Abdullah Ozer, John T. Lis, Harold G. Craighead

**Affiliations:** 1 School of Applied and Engineering Physics, Cornell University, Ithaca, NY 14853, USA; 2 Department of Molecular Biology and Genetics, Cornell University, Ithaca, NY 14853, USA

## Abstract

We describe a multiplexed RNA aptamer selection to 19 different targets simultaneously using a microcolumn-based device, MEDUSA (Microplate-based Enrichment Device Used for the Selection of Aptamers), as well as a modified selection process, that significantly reduce the time and reagents needed for selections. We exploited MEDUSA’s reconfigurable design between parallel and serially-connected microcolumns to enable the use of just 2 aliquots of starting library, and its 96-well microplate compatibility to enable the continued use of high-throughput techniques in downstream processes. Our modified selection protocol allowed us to perform the equivalent of a 10-cycle selection in the time it takes for 4 traditional selection cycles. Several aptamers were discovered with nanomolar dissociation constants. Furthermore, aptamers were identified that not only bound with high affinity, but also acted as inhibitors to significantly reduce the activity of their target protein, mouse decapping exoribonuclease (DXO). The aptamers resisted DXO’s exoribonuclease activity, and in studies monitoring DXO’s degradation of a 30-nucleotide substrate, less than 1 μM of aptamer demonstrated significant inhibition of DXO activity. This aptamer selection method using MEDUSA helps to overcome some of the major challenges with traditional aptamer selections, and provides a platform for high-throughput selections that lends itself to process automation.

Aptamers are short, often 30 to 100 nucleotides (nt) long, single-stranded nucleic acids with structures determined by their specific nucleotide sequence. These molecules are capable of binding with high affinity and specificity to a variety of targets ranging from small metal ions to membrane proteins on the surface of living cells^1^. Aptamers are usually discovered using a process called Systematic Evolution of Ligands by EXponential enrichment (SELEX) in which they are selected from a very large, sequence-diverse library of nucleic acids (10^12^–10^16^ unique sequences)^2^,^3^,^4^. SELEX is an iterative process, involving cycles of i) binding, where the target is incubated with the library; ii) partitioning, where the target-bound sequences are separated from the free sequences; and iii) amplification, where the target-bound sequences are amplified to create an enriched pool for the next round of selection. This process results in a final pool that is dominated by the strongest binding aptamer candidates. Aptamers are used in applications in a wide range of fields, including diagnostics, therapeutics, biotechnology, chemical analysis and separations^5^,^6^,^7^,^8^.

The standard SELEX process tends to be time consuming and uses a large amount of reagents making aptamer selections costly^9^,^10^. To reduce time and reagent consumption, we developed a modified version of SELEX we called RNA APtamer Isolation via Dual-cycles (RAPID)^11^. This method incorporates non-amplification cycles in which the eluted RNA is purified and used in a second binding cycle without prior amplification of the material. To compare the techniques, RAPID was performed in parallel with traditional SELEX using the same targets, and many of the same top aptamer candidates (~10% identical aptamers in the top 10,000 enriched sequences) emerged. Both selections using RAPID and traditional SELEX started with a library of 5 × 10^15^ unique sequences, yet the RAPID selection took approximately one third the time as our previously-optimized SELEX process and significantly reduced the amount of reagents used^11^. In this approach, only one non-amplification cycle was performed between amplification cycles, so it remained unknown whether multiple consecutive non-amplification cycles could be successful and further increase the number of total binding cycles possible within a given time.

Previously, we also developed a microcolumn technology designed for aptamer selections using an affinity chromatography approach. The target is immobilized on a resin packed within a microcolumn and the nucleic acid library is pumped through the column^12^. The scale of these devices (5–30 μL resin capacity) enabled a significant reduction in reagents used for a selection. Flow conditions were carefully optimized to yield the highest enrichment^12^. The microcolumns can also be connected in series allowing multiplex selections to be performed using a single aliquot of the valuable random library. In later cycles, selections are performed in parallel to avoid potential cross-contamination of enriched libraries^12^. One drawback of these devices is they are tedious to use in large-scale multiplex experiments. Consequently, we designed and fabricated a scaled-up version of the microcolumns for higher throughput. This device, named MEDUSA (Microplate-based Enrichment Device Used for the Selection of Aptamers), has all of the same benefits as the individual microcolumns, including reduced reagent consumption and the ability to be reconfigured between serial and parallel connections^13^. MEDUSA was also designed with specific dimensions and spacing that allow it to directly couple to a 96-well microplate. This enabled the continued use of high-throughput techniques in any downstream processing, which could be automated using existing liquid-handling robots. Similarly to individual microcolumn devices, we have optimized selection conditions, including protein density on the resin and flow-rates for aptamer library binding, washing, and elution, to yield the highest enrichment of previously-selected aptamers on their corresponding targets in a high-throughput manner^13^.

In this article, we describe the combination of the multi-well format MEDUSA device with the RAPID approach to simultaneously perform multiplexed aptamer selections to 19 different targets, including 4 background targets. The background targets, including maltose-binding protein (MBP), and amylose, nickel-nitrilotriacetic acid (Ni-NTA), and anti-FLAG resins, served as negative selections in the first round with the microcolumns connected in series. This allowed us to identify non-specific sequences that bound to these resins or the MBP-tag. The primary protein targets are associated with regulation of transcription, RNA stability, or localization. Mouse DXO and its yeast homolog (Ag Rai1) are decapping exoribonucleases that possess pyrophosphohydrolase, decapping and 5′-to-3′ exoribonuclease activities, and are thus implicated in RNA surveillance^14^,^15^. PNPase is a mitochondrial polynucleotide phosphorylase with 3′-to-5′ exoribonuclease and poly-A polymerase activities, and has recently been shown to be involved in the import of nuclear encoded RNAs into the mitochondrial matrix, thus indicating its involvement in mitochondrial homeostasis^16^,^17^. p23 is a molecular chaperone that initiates the disassembly of protein-DNA complexes, which impacts transcription factor activation potential and response time to environmental cues^18^. RTF1 is a subunit of the Paf1 complex (PAF1C), a multifunctional protein complex that associates with RNA Polymerase II and is implicated in histone modification, as well as transcriptional and posttranscriptional gene regulation^19^. NELF-E is a component of the NELF complex that negatively regulates elongation of transcription by RNA polymerase II^20^. The remaining 8 protein targets are various enzymes and enzymatic subunits involved in histone modification that are key features in epigenetic transcription regulation. These proteins are: TIP60-Chromo, GCN5-GNAT, MOF-Chromo, UTX-JMJC, JMJD2-Clav., JMJD2-JMJC, ASH1-BAH, and Trx-ZnFinger.

We exploited the reconfigurable design of the MEDUSA device to enable multiple selections to be performed using just 2 aliquots of library, and its microplate-based dimensions to perform downstream processes such as reverse transcription, PCR, quantitative PCR (qPCR), and transcription in a 96-well plate format. Encouraged by our earlier results with RAPID where amplification and non-amplification cycles are alternating, we decided to take our RAPID approach a step further by increasing the number of non-amplification cycles with later rounds of selection to drastically reduce the time and reagents needed for the selection. We performed the equivalent of a 10-cycle SELEX (with only 4 of these cycles requiring amplification) to 19 targets in about the time it would take to perform 4 cycles of conventional SELEX to one target. Analysis of high-throughput sequencing results allowed us to identify potential aptamer candidates, and many of these candidate aptamers were verified to bind the corresponding target proteins with a dissociation constant (K_D_) in the nanomolar range. In addition, further inspection of aptamer sequences enriched for DXO led us to identify aptamers that not only bind, but also resist DXO’s exoribonuclease activity. Furthermore, in studies monitoring the degradation of a 30-nt RNA substrate by DXO, the aptamers demonstrated significant inhibition of DXO activity. These and the aptamers to other proteins could be used for inhibiting target protein function, and the sequencing data provides a rich source of information to understand the biology of these target proteins and perhaps uncover novel functions (e.g. RNA interaction) and specificity.

## Results and Discussion

### MEDUSA as a platform for highly multiplexed aptamer selections

MEDUSA was designed for high-throughput aptamer selections and characterizations of the SELEX process using a microplate-based format allowing high-throughput downstream processes to be performed. This standardized format greatly increases the speed at which these processes are performed, and allows for potential automation of the aptamer selection process through the use of existing liquid-handling robots^21^,^22^. Multiplexing is especially beneficial with aptamer selections because it increases the likelihood of success per selection, where low success rates (30%) have been previously reported^9^,^23^. The SELEX process is also very time-consuming, often taking several weeks to months to complete^10^, so performing many selections simultaneously is extremely advantageous.

**Figure 1:**
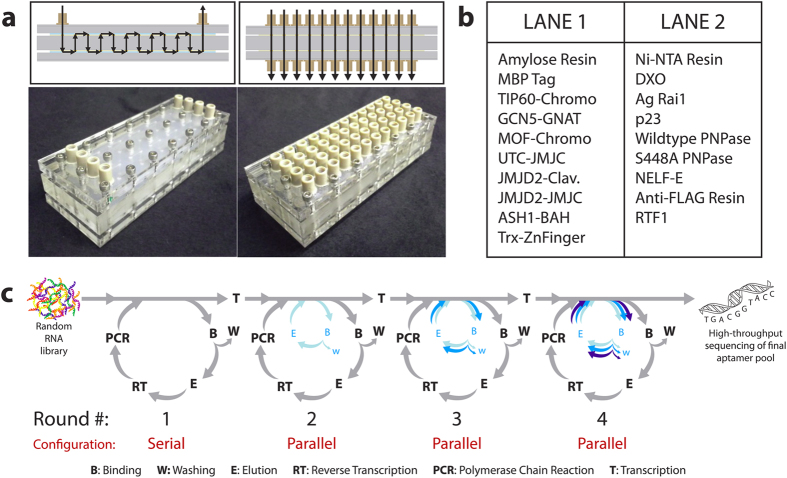
Schematics of the selection device and selection process. (**a**) The schematics describe the fluid path through the MEDUSA device in serial (left) and parallel (right) configurations, while the lower panels show images of the device in each configuration. (**b**) The list of targets in the order in which they saw the initial RNA library in a serial configuration. All the proteins in Lane 1 have an N-terminal MBP-tag, and in Lane 2 an N-terminal 6x His-tag, except RTF1, which has an N-terminal FLAG-tag for immobilization on amylose, Ni-NTA, and anti-FLAG resin, respectively. (**c**) The aptamer selection process beginning with a random RNA library, proceeding with 4 rounds of SELEX incorporating increasing numbers of non-amplification cycles prior to an amplification cycle, and ending with high-throughput sequencing of the enriched RNA pools. Ten cycles of selection were performed, starting at Round #1-Cycle #1 (R1C1) and ending at Round #4-Cycle #4 (R4C4).

MEDUSA is capable of connecting interchangeably between parallel and serially-connected microcolumns during a selection (Fig. 1a). This ability provides additional benefits, including the incorporation of in-line negative or counter selections and efficient use of the starting library. Here, just two aliquots of the random RNA library was used in selections to 19 different targets (Fig. 1b), which saved significant cost as the library is very expensive to synthesize. Additionally, performing aptamer selections using a microcolumn approach significantly reduces reagent consumption, most importantly the amount of target needed. This is beneficial when the target might only be available in limited quantity, especially for proteins that cannot be generated recombinantly in bacteria, but instead are purified from a mammalian cell line (e.g. FLAG-RTF1). Each cycle in these selections required just 6 μg of protein, which allowed selections to be carried out on protein targets that were difficult to purify in large quantities. Therefore, MEDUSA can provide advantages over other techniques that help to overcome some of the limitations frequently observed during aptamer selections.

### Incorporation of multiple non-amplification cycles to improve selection efficiency

Because the conventional SELEX process is time consuming, many approaches have been proposed to decrease the time required for a selection^9^,^10^. RAPID-SELEX was previously developed by our group, which incorporates a single non-amplification cycle between cycles that include amplification of the enriched aptamer pool^11^. Selections using RAPID-SELEX identified RNA aptamers in one third of the time compared to conventional SELEX. To further exploit the benefits of this process, additional non-amplification cycles were incorporated during the selections presented here. The selection consisted of 4 rounds ending in pool amplification, with a total of 10 binding cycles by incorporating increasing numbers of non-amplification cycles in each round (Fig. 1c). This allowed 10 binding cycles to be carried out in the same amount of time as 4 cycles of conventional SELEX, and with our process this took less than 4 weeks. This also dramatically reduced the cost of the selections by significantly reducing the amount of reagents used for reverse transcription, PCR amplification, and *in vitro* transcription. Including non-amplification cycles was also found to lead to a faster sequence convergence over traditional SELEX^11^.

### qPCR and high-throughput sequencing results

**Figure 2:**
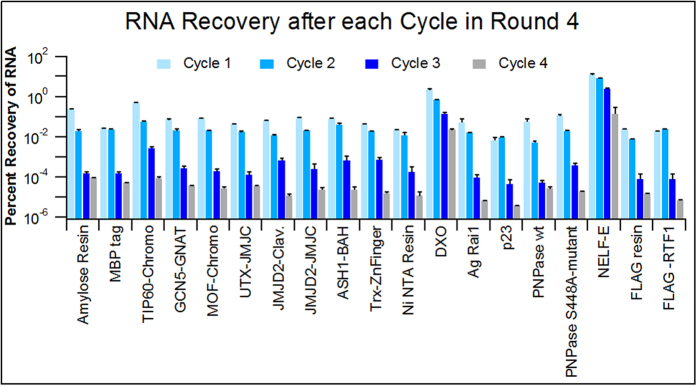
qPCR results indicating the percent recovery of RNA after each binding cycle in Round 4 relative to the initial RNA concentration at the beginning of the round. The error bars represent the standard deviation for triplicate measurements.

Quantitative PCR was used to measure the amount of RNA recovered after each binding cycle relative to the amount of RNA in the starting pool for each round. Figure 2 shows qPCR results measuring the percent recovery of RNA after each of the 4 cycles in Round 4 relative to initial concentration of RNA at the beginning of the round for all of the targets in this selection. All 19 targets showed the same trend with decreasing RNA recovery after each cycle. These results indicate that despite multiple consecutive binding cycles without amplification, a sufficient amount of RNA was recovered to warrant continuation of the selections. Since this trend was observed for every target, it can be concluded that this modified SELEX process can likely be used for any target. Furthermore, the targets with the highest affinity aptamers identified from the selection (DXO, TIP60-Chromo, and NELF-E) showed the highest RNA recovery during Round 4, suggesting that sequences with high affinity for these targets were being enriched.

To identify candidate aptamers for each target protein/domain and assess their enrichment, the final RNA pools [Round #4-Cycle #4 (R4C4)] and the pools after Round #3-Cycle #3 (R3C3) for every target were analyzed via high-throughput sequencing. All intermediate pools for four of the targets were also sequenced (Supplementary Table S1). This way, the presence of specific sequences can be monitored throughout the selection process, and help us better characterize the selection process with the MEDUSA device and modified RAPID-SELEX protocol. Unfortunately, of the four targets that were chosen for detailed analysis by high-throughput sequencing only one target, TIP60-Chromo, yielded sufficient aptamer convergence in the final and earlier rounds to be analyzed. Contrary to earlier reports, we did not observe that certain aptamers enriched in earlier cycles are replaced by other aptamers in later cycles (Supplementary Fig. S1)^24^. This difference could be a direct result of PCR-induced bias in conventional SELEX methods, where amplification of enriched pools is carried out after every cycle. Our RAPID-SELEX method greatly reduces the potential for such a bias, since amplification of enriched pools was carried out minimally, just 4 times in a 10-cycle selection.

Upon examination of the high-throughput sequencing results, sequences that could not be identified as an aptamer candidate (i.e. the constant regions were missing) were discarded, though a few still made it through our analysis pipeline (Supplementary Fig. S1). Sequences with 90% sequence similarity were grouped into clusters. The top 3,000 aptamer clusters for each target based on multiplicity in the final pools (R4C4) are provided in Supplementary Dataset 1. A total of 40 aptamer candidates for 8 of the 15 protein targets were selected for further testing of specific binding by Electrophoretic Mobility Shift Assay (EMSA). Five candidates were chosen for each target protein/domain based on having i) the highest multiplicity in the final RNA pool, ii) the highest enrichment from the R3C3 to the R4C4 pools, and iii) the highest specificity, in that these sequences were not highly abundant in any other RNA pool. The other 7 protein targets did not yield any candidate aptamers that were deemed appropriate for further testing, which would be sequences that had both high multiplicity and high specificity after 10 cycles. The selection was arbitrarily stopped after 4 rounds and 10 cycles because this was a proof-of-concept selection to demonstrate MEDUSA and the new RAPID approach. Additional cycles could be quickly performed, and would likely yield aptamers to more of these targets, as well as aptamers with improved affinity. The sequencing results highlight another advantage of multiplexing in that background binding or non-specific sequences can be easily identified and discarded from the pool of potential aptamer candidates. Previously, sequences that bind to the device and resins have been identified^13^, so these non-specific and other cross-reacting sequences can be identified and eliminated to save time and cost.

### Aptamer candidate testing

**Figure 3:**
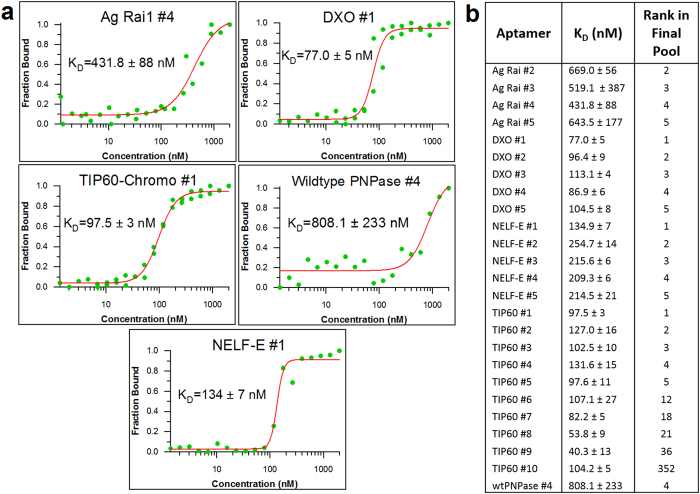
Binding analysis of candidate aptamer sequences. (**a**) Measurement of dissociation constants for aptamers to 5 different protein targets by EMSA experiments. The data were fitted to the Hill Equation to determine the K_D_. (**b**) A list of the dissociation constants for all of the aptamer candidates tested and their multiplicity ranking in their respective final (R4C4) pools. A complete list of the top 3,000 aptamer clusters for final pools of each target is provided in Supplementary Dataset 1.

EMSAs were performed to test the binding of candidate aptamers to their respective targets and determine the dissociation constants. Results for 5 of the aptamer candidates can be seen in Fig. 3a, as well as dissociation constants for all of the aptamer candidates tested that showed binding using this technique (Fig. 3b). A total of 25 aptamers were found for 5 different targets with dissociation constants in the nanomolar range (EMSA analyses shown in Supplementary Fig. S2), and it is likely that further optimization of the aptamer sequence would improve their affinity.

The copy numbers (multiplicity) of the selected aptamer candidates varied greatly, and did not predict whether the aptamer would bind nor did they correlate with the determined K_D_ (Fig. 3b). There were highly abundant sequences that did not bind to their targets, and sequences with only a few copies that did bind, so it is possible that more aptamers exist in the selected pools. From these results, we conclude that sequencing will indicate what sequences are present in the pool and their abundance, but it will not necessarily predict binding or affinity. High-throughput binding assays of target proteins to all aptamers could be used to provide a more comprehensive identification of the highest affinity aptamers in a selected pool^25^,^26^.

### Studying protein function through aptamer selection

**Figure 4:**
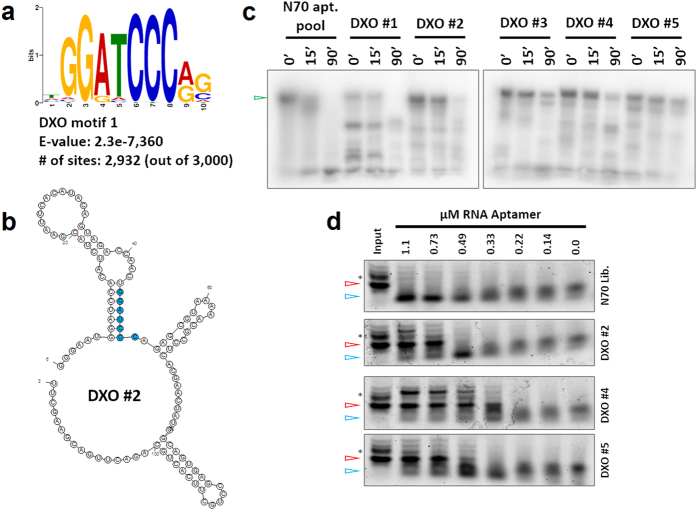
Characterization of DXO aptamers. (**a**) A highly specific DXO motif identified by MEME^27^,^28^ among the top 3,000 most abundant DXO aptamer clusters from R4C4. The E-value, representing the statistical significance of the identified motif as calculated by the MEME suite (http://meme-suite.org/), and number of aptamer clusters containing a match to Motif 1 are indicated. E-values lower than 0.05 are considered significant. (**b**) Secondary structure of DXO #2 aptamer, a representative aptamer of the second highest multiplicity cluster for DXO from the R4C4 pool, as predicted by the mFold Web Server (http://unafold.rna.albany.edu/?q=mfold/RNA-Folding-Form). DXO Motif 1 is colored cyan. Predicted secondary structures of other DXO aptamers are provided in Supplementary Fig. S3. (**c**) Stability of RNA aptamers against DXO exoribonuclease activity. Radiolabeled specific RNA aptamers or the N70 RNA library were incubated with DXO enzyme at 37 °C for the indicated times and separated using 8% denaturing PAGE. Full-length RNA aptamers are indicated by 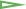. (**d**) Inhibition of DXO exoribonuclease activity by RNA aptamers. Degradation of a 3′-Cy5-labeled 30-nt RNA substrate by DXO in the absence and presence of indicated amounts of RNA aptamers as determined by 10% denaturing PAGE analysis. Full-length, intact RNA substrate and degradation product are indicated by 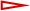 and 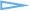, respectively. The band indicated by * above the full-length RNA is likely incompletely deprotected RNA substrate (2′-ACE protected, Dharmacon).

To gain further insight on the selection process and how it could be used to study the biological function of a target protein, as well as provide useful tools that do more than just bind, we turned our focus to the DXO protein, which has 5′-3′ exoribonuclease and is implicated in RNA surveillance^14^,^15^. To identify short RNA motifs that are enriched in DXO candidate aptamers, we subjected the random regions of the top 3,000 most abundant aptamer clusters from the DXO R4C4 pool to MEME analysis^27^,^28^. This analysis led to identification of DXO Motif 1 (5′-GGATCCC-3′), which showed significant enrichment (E-value: 2.3e-7,360), was present in almost every candidate aptamer (2,932 out of 3,000), and showed high-specificity for DXO in that it was not identified in similar MEME analysis of other target pools (Fig. 4a). The specificity of DXO aptamers containing this motif was also tested, and these aptamers showed no appreciable binding to non-specific targets (Supplementary Fig. S3). This motif is similar to the BamHI restriction site, which is included in the forward constant region of the N70 RNA library (see Materials and Methods). The motif is found to base-pair with the BamHI site in secondary structure predictions giving rise to a stable stem structure close to the 5′-end of the RNA aptamer (Fig. 4b and Supplementary Fig. S4). We reasoned this may hinder the 5′-3′ exoribonuclease activity of DXO. We tested this hypothesis by incubating radiolabeled RNA aptamers (5 DXO candidate aptamers and the N70 RNA library) with DXO at 37 °C in DXO exoribonuclease buffer, and monitoring the stability of full-length RNA via denaturing (7 M urea) polyacrylamide gel electrophoresis (PAGE) analysis. As shown in Fig. 4c, all five candidate DXO aptamers showed resistance against DXO exoribonuclease activity, with DXO #3, #4, and #5 showing the strongest resistance, when compared to the N70 RNA library control. Using a finer time-course for these tests, we estimated the half-lives of the DXO candidate aptamers to be ~1.5-3.5-fold higher (28.9–69.3 min) than that of the N70 library (19.8 min) (Supplementary Fig. S5). DXO has significantly lower exoribonuclease activity in SELEX buffer compared to DXO exoribonuclease buffer (data not shown), perhaps explaining why these aptamers were enriched and survived incubations with DXO for as long as 17 hours.

To test whether the determining factor for RNA aptamer resistance against DXO exoribonuclease activity is the sequence of the motif itself or just the stability of the first stem in the aptamer structure, we took the DXO #2 and #5 aptamers with perfect matches to DXO Motif 1, and generated mutant aptamers. In mut1 aptamers, the sequence of the motif was altered but the stability was maintained by changing the original motif (5′-GGATCCC-3′ to 5′-CCTAGGC-3′) and the BamHI site in the forward constant region (5′-GGATCC-3′ to 5′-CCTAGG-3′). In mut2 aptamers, the stability of the first stem was reduced by changing the original motif to 5′-AATATTC-3′ and the BamHI site in the forward constant region to 5′-AATATT-3′. In addition, we generated truncations of DXO #2 and #5 aptamers, which retained the DXO Motif 1 and the first stem-loop structure for each aptamer. A sequence-shuffled version of DXO #5 trunc was used as a control. As shown in Supplementary Fig. S6, these mutant and truncated aptamers are predicted to adopt very similar, if not identical, secondary structures to the original aptamers. In the DXO exoribonuclease assay, the mut1 and truncated aptamers showed nearly identical stabilities compared to the original full-length RNA aptamers, whereas the mut2 aptamers showed enhanced susceptibility to degradation by DXO, similarly to that of the N70 aptamer library and DXO #5 shuffle control (Supplementary Fig. S6). These results highlight the importance of an RNA molecule’s 5′-end stability in determining its susceptibility to DXO-mediated degradation, which sheds light on the *in vivo* function of DXO and can help to identify *in vivo* targets of DXO exoribonuclease activity.

Finally, we tested whether these RNA aptamers could inhibit the exoribonuclease activity of DXO. We measured the exoribonuclease activity on a 30-nt RNA substrate that was 3′-Cy5 labeled. DXO was pre-incubated with a range of concentrations (0–1100 nM) of full-length RNA aptamers, and the RNA substrate was subsequently added. As shown in Fig. 4d, even in the presence of 1.1 μM N70 RNA library, the Cy5-labeled substrate was almost completely degraded. Conversely, a substantial amount of RNA substrate was still intact after 2 hours of incubation with 1 μM DXO in the presence of 730 nM DXO #2, 330 nM DXO #4, and 490 nM DXO #5 aptamer. By fitting these data to a dose-response curve, we calculated ≫1100 nM, ~610 nM, ~290 nM, and ~570 nM IC50 values for the N70 RNA library, DXO #2, DXO #4, and DXO #5 aptamers, respectively (Supplementary Fig. S7). In these samples, we have verified equal loading of different aptamers and the library, and they remained largely intact (Supplementary Fig. S7). Truncated versions of DXO #2 and #5 aptamers did not inhibit DXO exoribonuclease activity (Supplementary Fig. S8). These results suggest that the full-length DXO aptamers, but not the truncated versions, inhibit the exoribonuclease activity directly, not simply by competing off the RNA substrate.

Given their moderately-high affinity and DXO exoribonuclease inhibitory effect, these full-length aptamers could potentially be used as DXO inhibitors for studying DXO function *in vivo*. In the absence of a highly specific small molecule inhibitor of DXO, DXO-specific inhibitory RNA aptamers, which can in principle be expressed transgenically at specified levels, time points, and durations^29^,^30^, enable the functional study of DXO exoribonuclease activity *in vivo*. Likewise, compared to RNAi knockdowns, inhibitory RNA aptamers offer unique advantages for the following *in vivo* functional studies: i) a specific function or interaction of the target protein (e.g. exoribonuclease activity of DXO) could be targeted rather than complete elimination of the entire protein as with RNAi, and ii) distinction between primary and secondary effects is possible, since inhibitory concentrations of RNA aptamers could be attained relatively quickly (i.e. minutes to tens of minutes) upon induction, whereas significant knockdown of most proteins by RNAi requires hours to days.

These studies have shown that different properties of the target proteins can also be discovered and studied through an aptamer selection. Furthermore, selecting sequences for more than just affinity, such as nuclease resistance and inhibition, can be accomplished. Some of the targets in this selection possess nuclease activity, which affected the affinity testing, and likely the selection process itself, as sequences with a higher resistance to the nuclease activity would be selected over those that may have higher affinity. DXO possesses 5′-3′ exoribonuclease activity, and we have shown that the selected aptamers resist degradation significantly longer than the random RNA library (Fig. 4a,b). We also demonstrated that this resistance is due to the physical structure of the aptamer with a stem loop at the 5′ end. This type of structure was also shown to inhibit the exoribonuclease activity against an RNA substrate meant to represent nascent RNA (Fig. 4c). These results demonstrate an additional benefit and application of the SELEX process.

## Conclusions

We have demonstrated that a highly multiplexed aptamer selection can be performed using the MEDUSA device, and its reconfigurable microcolumn design allows for substantial reduction in reagent consumption. Most notably, a single aliquot of the starting random library can be used to select aptamers to multiple different targets, and just 6 μg of protein target is needed for each cycle. Additionally, a modified SELEX process incorporating increasing numbers of consecutive non-amplification cycles significantly reduced the time required to perform the selections. Most importantly, high-throughput aptamer selections are particularly beneficial because they increase the rate of success per selection.

High-throughput sequencing allows for the identification of top sequences, as well as their multiplicity in the pool. Performing multiplex aptamer selections is also beneficial when analyzing sequencing results, since background binding and non-specific sequences can be recognized when they appear in multiple pools with significant abundance. However, after identifying potential aptamer candidates and performing binding experiments, it was obvious that sequence multiplicity could not predict binding or the affinity of the aptamer.

Finally, through these selections, we demonstrated that useful tools, other than high-affinity ligands, can be discovered. Aspects of a target protein’s function were studied, and aptamers capable of resisting exoribonuclease activity and acting as inhibitors were determined. Therefore, aptamer selections can produce very useful tools that can be used in many different applications, and the device and selection process presented here provides advantages over other processes that improve the efficiency and success of aptamer selections.

## Materials and Methods

### Target protein preparation

MBP-tagged protein domains were purified from *E. coli* Bl21 (DE3) RIPL strain using amylose resin (NEB) according to the manufacturer’s recommendations. Briefly, for each protein the bacteria were grown to an OD600 of 0.6 in 1 L of LB media supplemented with 100 μg/mL of ampicillin, and induced with 200 μM IPTG. Induction cultures were then incubated at 18–22 °C for 12–14 hours with 250 rpm shaking. Bacteria pelleted at 3500 rpm and 4 °C for 30 min were lysed in MBP buffer (20 mM Tris-Cl pH 7.5, 300 mM NaCl, 1 mM EDTA, 5 mM BME) supplemented with 0.1% NP-40, 1 mg/mL Lysozyme, and 1X Protease Inhibitor Cocktail (Roche). After a 15-min incubation on ice, lysates were sonicated on ice 3 times for 30 s each using a Branson Cell Distributor with a microtip, and cleared by centrifugation at 20,000 × g and 4 °C for 45 min. Cleared lysates were applied to 1 mL of pre-washed amylose resin and allowed to pass through the column under gravity flow. Protein-bound resin was washed 3 times with 10 mL of MBP buffer, and the proteins were eluted in five 1-mL fractions using MBP buffer supplemented with 10 mM maltose. Protein concentration was measured by Bradford Protein Assay (Bio-Rad) against a BSA standard, and the purity of each protein preparation was determined by SDS PAGE and Coomassie Blue staining. One or two fractions that had the highest protein concentration (often >1 mg/ml) and were relatively pure (>80% pure estimated from Coomassie stained gel) were supplemented with 10% glycerol, aliquoted, and stored at −80 °C. The maltose used for elution was not dialyzed, since most proteins precipitated out during dialysis, and re-binding of these proteins was done at very dilute conditions (>100-fold dilution, thus <0.1 mM maltose), which permitted efficient re-binding of the purified proteins to the amylose resin for the aptamer selections.

Hexahistidine (6xHis)-tagged NELF-E was purified as described previously^20^. All other target proteins were kindly provided by others: 6xHis-tagged DXO and Ag Rai1 by the Tong Lab at Columbia University, 6xHis-tagged wildtype and S448A mutant versions of PNPase by the Teitell Lab at the University of California, Los Angeles, 6xHis-tagged p23 by the Freeman Lab at the University of Illinois, and FLAG-tagged RTF1 by the Marazzi Lab at Mount Sinai Graduate School for Biomedical Sciences. Protein concentrations ranged between 0.3 mg/mL (FLAG-RTF1) and 34 mg/mL (PNPase). Supplementary Table S2 contains detailed information for all target proteins and tags used in the MEDUSA selections.

### Target protein immobilization on affinity resins

For each cycle of the selection, fresh protein-immobilized resins were prepared. Amylose, Ni-NTA, and anti-FLAG resins were washed thoroughly with binding buffer (25 mM Tris-Cl pH 7.5, 100 mM NaCl, 25 mM KCl, and 1 mM MgCl_2_) prior to target immobilization. MBP-, 6xHis-, and FLAG-tagged protein targets were immobilized onto amylose, Ni-NTA, and anti-FLAG resins, respectively, by incubating the proteins at a concentration of 0.6 μg/μL of resin with the resin in a 10% slurry in binding buffer. The target resins were incubated for 1 h at 4 °C with constant mixing. A list of targets and the order in which they were connected in series for the first round of selection can be found in Fig. 1b.

### Library and primers

The random RNA library used in this selection contained ~5 × 10^15^ RNA sequences of 120 nt in length, which was transcribed *in vitro* from a DNA library that was chemically synthesized by GenScript. The RNA library consisted of a 70-nt random region flanked by two constant regions (5′-GGGAAUGGAUCCACAUCUACGAAUUC- N_70_ -UUCACUGCAGACUUGACGAAGCUU-3′) as described elsewhere^21^. The forward and reverse primers used for PCR, qPCR, and reverse transcription were T7pro-AptLibConsFOR, 5′-GATAATACGACTCACTATAGGGAATGGATCCACATCTACGA-3′, and AptLibConsREV, 5′-AAGCTTCGTCAAGTCTGCAGTGAA-3′, respectively. The forward oligo contains the T7 promoter sequence (underlined) used for transcription of the DNA template to RNA, and a shortened version of the original forward constant region to reduce primer dimer formation. These primers and other oligos, including the gBlock templates of the candidate aptamers, were purchased from Integrated DNA Technologies.

### MEDUSA fabrication

A description of MEDUSA’s components, as well as its fabrication, is described in detail elsewhere^13^. Briefly, a MEDUSA consisting of 48 microcolumns arranged in a 4 × 12 grid (Fig. 1a) was fabricated using a CO_2_ laser at 10.6 μm (Universal Laser Systems, VersaLaser) to cut the poly(methyl methacrylate) (PMMA) and silicone layers. Each microcolumn had a 10-μL volume capacity, and fluidic connections were made using standard microfluidic connectors purchased from IDEX. This device was assembled such that it could be reversibly configured between the microcolumns connected in series or in parallel. Between rounds of selection, the device was washed extensively with 1 M KOH, 1 M HCl, and 1x binding buffer separated by DEPC water washes, and reused for all subsequent rounds.

### RNA aptamer selection process

All solutions were degassed and introduced into the device via a standard syringe pump (Harvard Apparatus). The selection consisted of 4 rounds ending in pool amplification, with a total of 10 binding cycles by incorporating increasing numbers of non-amplification cycles in each round (Fig. 1c). The flow parameters used in each round of the selection are summarized in Table 1.

In the first round of selection, MEDUSA was configured with microcolumns connected in series, and two aliquots of the N70 RNA library were used for 19 total targets. The order of target proteins in each series is shown in Fig. 1b. The target-immobilized resins were loaded into MEDUSA using a pipette, with 10 μL of resin in each microcolumn. With MEDUSA connected in parallel to prevent target contamination, the resin-packed microcolumns were washed and primed by flowing 1 mL of binding buffer at 100 μL/min, which removed any unbound proteins from the microcolumns. The random RNA library was diluted to 1 mL in binding buffer, and heat denatured at 65 °C for 5 min. The library was allowed to slowly cool to room temperature while degassing, and finally 200 units of RNase inhibitor (SUPERase•In, Ambion) were added to each library. A 10-μL sample of each library was collected and used as a standard for qPCR analysis. MEDUSA was reconnected in series for the binding step, and the libraries were pumped at 1 μL/min in the first round. MEDUSA was then reassembled into a parallel configuration for the remaining selection steps. After RNA library binding, each microcolumn was washed with 3 mL of binding buffer at 1 mL/min. To elute the protein-RNA complexes from each microcolumn, 400 μL of elution buffer [100 mM ethylenediaminetetraacetic acid (EDTA) (for Ni-NTA resin), 50 mM maltose (for amylose resin), and 100 μg/mL 3x FLAG peptide (for anti-FLAG resin) in binding buffer] were pumped at 50 μL/min, and the eluent was collected in a 96-well microplate coupled to MEDUSA. The eluted RNA was purified by phenol-chloroform extraction, and concentrated via ethanol precipitation with 1 μL of GlycoBlue (Ambion) and 40 μg of yeast tRNA (Invitrogen). The resulting pellet was resuspended in RNase-free water. This RNA pool subsequently underwent either another binding reaction (non-amplification cycle), or immediate reverse-transcription and PCR amplification.

The standards and resuspended pools were reverse transcribed using Moloney Murine Leukemia Virus Reverse Transcriptase (MMLV-RT). Residual RNA was digested using a cocktail of RNases, including RNase H, A, and T1 (Invitrogen). Approximately 3% of the cDNA from each pool was used to determine the RNA recovery compared to the initial quantity of RNA at the beginning of the round via qPCR. The remaining cDNA was PCR-amplified, and purified using DNA Clean & Concentrator spin columns (Zymo Research). A fraction of the purified DNA was used for transcription with T7 RNA polymerase (Invitrogen) to produce the RNA pools for the next round of selection. Following transcription, the template DNA was digested using DNase I (Ambion), and the RNA was phenol-chloroform extracted and ethanol precipitated. The purified RNA pools were verified by denaturing PAGE for purity and length, and quantified via Qubit BR RNA assay (Invitrogen).

In rounds 2, 3 and 4 of the selection, 50 μg of the enriched RNA pools were used, and an increasing number of non-amplification cycles were incorporated in which the eluted RNA was purified by phenol-chloroform extraction, ethanol precipitated, and directly subjected to the next binding cycle without amplification. The flowrates were increased slightly to allow multiple binding cycles to be performed within a single day (see Table 1).

### High-throughput sequencing of pools

A small amount of cDNA from each final pool was PCR-amplified using primers containing unique 6-nt barcodes and the necessary adapters for the HiSeq 2500 (Illumina) sequencing platform operated in Rapid Run Mode. The sequencing data was analyzed with readily available software. Briefly, the sequencing data was quality filtered, barcode split, trimmed of constant regions and barcodes, and collapsed using programs included in the FASTX toolkit (http://hannonlab.cshl.edu/fastx_toolkit/). The resulting library of sequences was first clustered using UCLUST software, part of the USEARCH software^31^, at a 90% sequence identity threshold. To identify the abundance of all aptamer clusters in every target library, the original aptamer clusters were then reclustered as before, and the total number of sequences in each cluster in every target library was tabulated. Only a representative aptamer sequence, the most abundant sequence in a given cluster, is listed for a cluster. Sequences with sufficient multiplicity (defined as: number of sequences in a cluster/number of total sequences in the library), enrichment (defined as: abundance in the last round or cycle/abundance in an earlier round or cycle), and specificity (defined as: abundance in the target-specific library/abundance in all other libraries) were identified as probable aptamer candidates.

### Motif search using MEME

To identify motifs, 5–20 nt RNA sequences that were enriched in a selected RNA pool and present in the top 3000 aptamer clusters were subjected to analysis by the MEME program of the MEME Suite (http://meme-suite.org/)^27^,^28^. The E-value, representing the statistical significance of the identified motif, is estimated by MEME as the log likelihood ratio of finding an identical motif with the same width and site count in a similarly sized set of randomized sequences with identical base composition. Small E-values (up to 0.05) are considered significant, with smaller ones being more significant. Motifs identified by MEME for target proteins were found to be specific and not identified in other target pools.

### Binding affinity determination via EMSA

To test the binding capability of the aptamer candidates and determine the dissociation constant, EMSAs were performed for each candidate. The RNA aptamer candidate was fluorescently labeled at the 3′ end with fluorescein isothiocyanate (FITC)^32^. With the quantity of RNA constant, a series of protein concentrations were incubated with the labeled aptamer candidate for 60 minutes. These samples were then run at 4 °C on an agarose gel (1.5% agarose, 0.5x TBE, 1 mM MgCl_2_), and imaged using a fluorescence scanner. The gel images were quantified using ImageJ, and the resulting curves were fitted to the Hill Equation using Igor Pro 5.04A to determine the K_D_. For many of the aptamers, a second EMSA was performed, and the data from both experiments were merged and fitted to the Hill Equation.

### Analysis of DXO aptamers resisting exoribonuclease activity and acting as inhibitors

Body-labeled RNA aptamers were generated by *in vitro* transcription with T7 RNA polymerase and [α-P^32^]UTP (Perkin Elmer), and purified by RNase-free Micro Bio-Spin P-30 columns (Bio-Rad). Exoribonuclease assays were performed as described elsewhere^15^. Briefly, radiolabeled RNA aptamers (10,000 cpm in RNase-free DXO buffer: 50 mM NaCl, 5 mM MgCl_2_, 20 mM Tris-Cl pH 8.0, 0.5 mM DTT, 0.025 mg/mL UltraPure BSA) were first heat-denatured at 70 °C for 5 min and renatured at room temperature for 15–30 min. They were then incubated with 1 μM recombinant DXO enzyme (in DXO buffer) for various durations at 37 °C. For testing the inhibition of DXO 5′-3′exoribonuclease activity by RNA aptamers, DXO was first equilibrated with RNA aptamers at room temperature for 5 min. 3′-Cy5-labeled 30-nt RNA substrate (Dharmacon)^33^ was then added, and the reaction was incubated at 37 °C for 2 hours. The final reactions contained 1 μM DXO, 20 nM 3′-Cy5-labeled RNA substrate, and various concentrations of DXO RNA aptamers (0–1100 nM) in DXO buffer. After incubation, each reaction was mixed with an equal volume of 2X Gel Loading Buffer (Ambion), heat denatured at 75 °C for 5 min, and run on 8 or 10% denaturing polyacrylamide gels. After gel running, the gel was either dried on Whatman paper, exposed to a Phosphorimager screen, and the screen was imaged using a Typhoon 9400 scanner, or the gel was EtBr stained and imaged with a Typhoon 9400 scanner. Quantification of these images was performed using ImageJ software, and the data analysis was carried out either in Microsoft Excel or in GraghPad Prism6 software.

## Additional Information

**How to cite this article**: Reinholt, S. J. *et al*. Highly Multiplexed RNA Aptamer Selection using a Microplate-based Microcolumn Device. *Sci. Rep.* **6**, 29771; doi: 10.1038/srep29771 (2016).

## Supplementary Material

Supplementary Information

Supplementary Dataset 1

## Figures and Tables

**Table 1:** Selection Parameters.

Round	Number of Binding Cycles	Binding Step Parameters	Washing Step Parameters	Elution Step Parameters
1	1	1 mL at 1 μL/min	3 mL at 1 μL/min	400 μL at 50 μL/min
2	2	1 mL at 10 μL/min	1 mL at 1 mL/min, 2 mL at 70 μL/min	400 μL at 50 μL/min
3	3	1 mL at 10 μL/min	1 mL at 1 mL/min, 2 mL at 70 μL/min	400 μL at 50 μL/min
4	4	1 mL at 10 μL/min	1 mL at 1 mL/min, 2 mL at 70 μL/min	400 μL at 50 μL/min
