# Supplementary material for: Highly Multiplexed RNA Aptamer Selection using a Microplate-based Microcolumn Device
Source: Sci Rep. 2016 Jul 19;6:29771. doi: 10.1038/srep29771 (PMC4949599; doi:10.1038/srep29771)
Supplement: Supplementary Information [file srep29771-s1.pdf]

Supplementary Information

Highly multiplexed RNA aptamer selection using a microplate-based microcolumn device

Sarah J. Reinholt, Abdullah Ozer, John T. Lis and Harold G. Craighead

Supplementary Table S1: Summary of sequencing results and clustering

	**Total # of reads**	**Quality filtered (>Q25 for 80%)**	**Percent passed quality filter:**			
Lane1	210774447	132780375	63.00			
Lane2	205393613	68306006	33.26			
**Sequencing Lane**	**Target protein**	**SELEX Round-Cycle**	**Barcode**	**Barcode matched**	**Clustered Reads**	**% Clustered**
Lane1	Amylose	R3C3	1	3751360	3090917	82.4
		R4C4	2	4275552	3860899	90.3
	GCN5_GNAT	R2C2	3	3632234	3335381	91.8
		R3C2	4	3315571	2993167	90.3
		R3C3	5	3347685	3025012	90.4
		R4C2	6	3454226	3127458	90.5
		R4C3	7	4888895	4364730	89.3
		R4C4	8	4086807	3535763	86.5
	JMJD2_Clav	R3C3	9	2444505	2246627	91.9
		R4C4	10	5013347	4200451	83.8
	MBP	R2C1	11	2264022	2092324	92.4
		R2C2	12	4830747	4401192	91.1
		R3C2	13	4505792	4081211	90.6
		R3C3	14	4782592	4367501	91.3
		R4C2	15	4108004	3648420	88.8
		R4C3	16	3453035	3103763	89.9
		R4C4	17	2194247	1922155	87.6
	MOF_Chromo	R3C3	18	3983548	3664643	92.0
		R4C4	19	4043852	3598491	89.0

	TIP60_Chromo	R2C1	20	4093407	3735109	91.2
		R2C2	21	3930809	3544290	90.2
		R3C2	22	3575983	3266581	91.3
		R3C3	23	2406272	1641927	68.2
		R4C2	24	3729753	3104503	83.2
		R4C3	25	3144953	2892011	92.0
		R4C4	26	4582331	4132911	90.2
	UTX_JMJC	R3C3	27	5184360	4734991	91.3
		R4C4	28	3681639	3297236	89.6
	Unmatched			18190321		
Lane2	AgRai1	R3C3	1	2324032	2146812	92.4
		R4C4	2	84290	76828	91.1
	Ash1_BAH	R3C3	3	2269338	2102474	92.6
		R4C4	4	1884865	1611908	85.5
	DXO	R3C3	5	2211899	2040984	92.3
		R4C4	6	2090182	1951136	93.3
	FLAG-RTF1	R3C3	7	2131522	1961767	92.0
		R4C4	8	1289219	1124605	87.2
	FLAG	R3C3	9	2365489	2185911	92.4
		R4C4	10	2173980	1925590	88.6
	JMJD2_JMJC	R3C3	11	2575224	2383706	92.6
		R4C4	12	1102417	983567	89.2
	NELF-E	R2C1	13	2486073	2318500	93.3
		R2C2	14	2347505	2172774	92.6
		R3C2	15	1731767	1590004	91.8
		R3C3	16	1135320	1038583	91.5
		R4C2	17	2132048	1973489	92.6
		R4C4	18	2194432	2035697	92.8
	NiNTA	R3C3	19	1584669	1432636	90.4
		R4C4	20	5926827	5306374	89.5

	Trx_ZnF	R3C3	21	3008680	2753132	91.5
		R4C4	22	2844793	2529447	88.9
	mutPNPase	R3C3	23	1756627	1622867	92.4
		R4C4	24	1830710	1683166	91.9
	wtPNPase	R3C3	25	481140	338727	70.4
		R4C4	26	919315	749263	81.5
	p23	R3C3	27	2672349	2467321	92.3
		R4C4	28	2517795	2308017	91.7
	unmatched			7046111		

Supplementary Table S2: Target Protein Information

Protein Name	GenBank ID	Protein Function	Specific Domain	Domain Boundaries (a.acids - a.acids)	Domain Function	Molecular Weight (kDa)	Isoelectric point (pI)	Affinity Tag/Resin used for SELEX
TIP60	NM_131923	H4 and H2A histone acetyltrasnferase. Catalytic subunit of NuA4 HAT complex.	Chromodomain	11-300	Involved in protein-protein and/or protein-nucleic acid interactions. Many chromodomains act as methyl-specific histone binding module, and some chromodomains were found to associate with RNA.	31.5	8.25	MBP/ Amylose
GCN5	AF029776	A major histone acetyltransferase, which promotes transcriptional activation. Catalytic subunit of SAGA, SALSA, and ADA complexes.	GNAT domain	481-627	Gcn5-related N-acetyltransferases (GNAT) catalyze the transfer of the acetyl from the CoA donor to a primary amine of the acceptor.	16.8	8.64	MBP/ Amylose
MOF	NM_078496	A major histone acetyltransferase, which promotes transcriptional activation. Catalytic subunit of MSL and NSL complexes.	Chromodomain	376-539	Involved in protein-protein and/or protein-nucleic acid interactions. Many chromodomains act as methyl-specific histone binding module, and some chromodomains were found to associate with RNA.	18.7	7.00	MBP/ Amylose
UTX	NM_135524	Histone demethylase that specifically demethylates 'Lys-27' of histone H3.	JMJC domain	832-995	Catalytic domain responsible for histone demethylation. Predicted metalloenzyme domain with cupin fold that binds Fe(III) and alphaKG.	18.7	7.15	MBP/ Amylose
JMJD2	NM_136487	Histone demethylase that specifically demethylates 'Lys-9' and 'Lys-36' residues of histone H3.	Clavaminate synthase domain	95-336	Catalytic domain responsible for histone demethylation. Predicted metalloenzyme domain with cupin fold that binds Fe(III) and alphaKG.	28.7	8.33	MBP/ Amylose

JMJD2	NM_136487	Histone demethylase that specifically demethylates 'Lys-9' and 'Lys-36' residues of histone H3.	JMJC domain	149-315	Catalytic domain responsible for histone demethylation. Predicted metalloenzyme domain with cupin fold that binds Fe(III) and alphaKG.	19.5	6.32	MBP/ Amylose
ASH1	NM_079436	Trithorax group (TrxG) protein with histone methyltransferase activity, which specifically trimethylates 'Lys-4' of histone H3 (a specific tag for epigenetic transcriptional activation) and to a lesser extend trimethylate H3 'Lys-9' and H4 'Lys-20'.	BAH domain	1943-2063	BAH (bromo-adjacent homology) is commonly found in chromatin-associated proteins implicated in transcriptional regulation. Acts as protein-protein interaction modules.	14.5	8.26	MBP/ Amylose
TrX	NM_134282	Histone methyltransferase responsible for methylating 'Lys-4' of histone H3 (a specific tag for epigenetic transcriptional activation). Functions in segment determination through interaction with genes of bithorax (BX-C) and antennapedia (ANT-C) complexes.	Zinc Finger	1266-1482	Plant homeodomain (PHD) zinc finger domain has a C4HC3-type motif, found in many chromatin regulatory factors. They can bind zinc and other metal ions. Involved in protein-protein and protein-DNA interactions, and impliaceted in transcriptional regulation, translation, chromatin remodelling and etc.	24.3	8.39	MBP/ Amylose
MBP tag (MalE)	AF097412	Affinity tag	N/A	1-400	Maltose binding / Affinity tag	44.0	4.88	N/A

	GenBank ID	Protein Function	Molecular Weight (kDa)	Isoelectric point (pI)	Affinity Tag / Resin used for SELEX	Reference PubMed ID
NELF-E	NM_139984	Regulation of RNA Pol II elongation, maintenance of paused RNA Pol II.	31.8	9.47	6xHis / Ni-NTA	24453987
DXO	NM_033613	Exoribonuclease that specifically degrades pre-mRNAs with a defective 5'-cap and is part of a pre-mRNA capping quality control. Has decapping, pyrophosphohydrolase and 5'-3' exonuclease activities.	45.3	8.29	6xHis / Ni-NTA	23523372
Ag Rai1	NM_001181112	Exoribonuclease that specifically degrades pre-mRNAs with a defective 5'-cap and is part of a pre-mRNA capping quality control. Has decapping, pyrophosphohydrolase and 5'-3' exonuclease activities.	44.5	6.14	6xHis / Ni-NTA	19194460

p23	NM_001179683	Molecular chaperone implicated in gene regulation by disassembly of protein-DNA complexes, which in turn allows GCN5 acetyltransferase to prolong the dissociated state through lysine acetylation.	24.1	4.46	6xHis / Ni-NTA	23022381
PNPase (wildtype and S484A mutant)	NM_033109	Polyribonucleotide nucleotidyltransferase 1 catalyzes the phosphorolysis of RNA processively in the 3'-to-5' direction as part of the mitochondrial degradosome (mtEXO) complex, also implicated in translocatio of nuclear-encoded RNA into mitochindria matrix.	86.0	7.87	6xHis / Ni-NTA	20691904
6xHis tag	AF097413	Ni+2 binding / Affinity tag	3.7	5.42		
	**GenBank ID**	**Protein Function**	**Molecular Weight (kDa)**	**Isoelectric point (pI)**	**Affinity Tag / Resin used for SELEX**	**Reference PubMed ID**
RTF1	NM_015138	Component of the PAF1 complex (PAF1C) which has multiple functions during transcription by RNA polymerase II and is implicated in regulation of development and maintenance of embryonic stem cell pluripotency.	80.3	8.21	FLAG peptide / FLAG M2 resin	26217014
Flag peptide tag	N/A	FLAG M2 antibody binding / Affinity tag	2.7	3.97	N/A	N/A

a

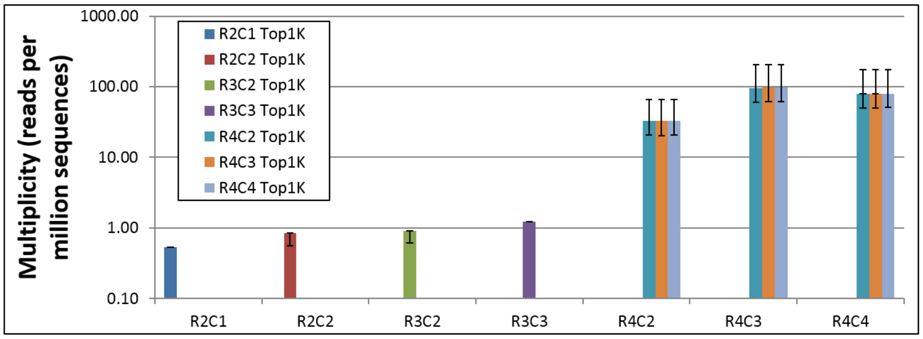

b

Common aptamers between Tip60-Chromodomain selected pools							
	**R2C1**	**R2C2**	**R3C2**	**R3C3**	**R4C2**	**R4C3**	**R4C4**
**R2C1**	1000	4	4	3	1	1	2
**R2C2**		1000	6	5	3	4	5
**R3C2**			1000	3	3	4	4
**R3C3**				1000	21	21	22
**R4C2**					1000	906	905
**R4C3**						1000	933
**R4C4**							1000

c

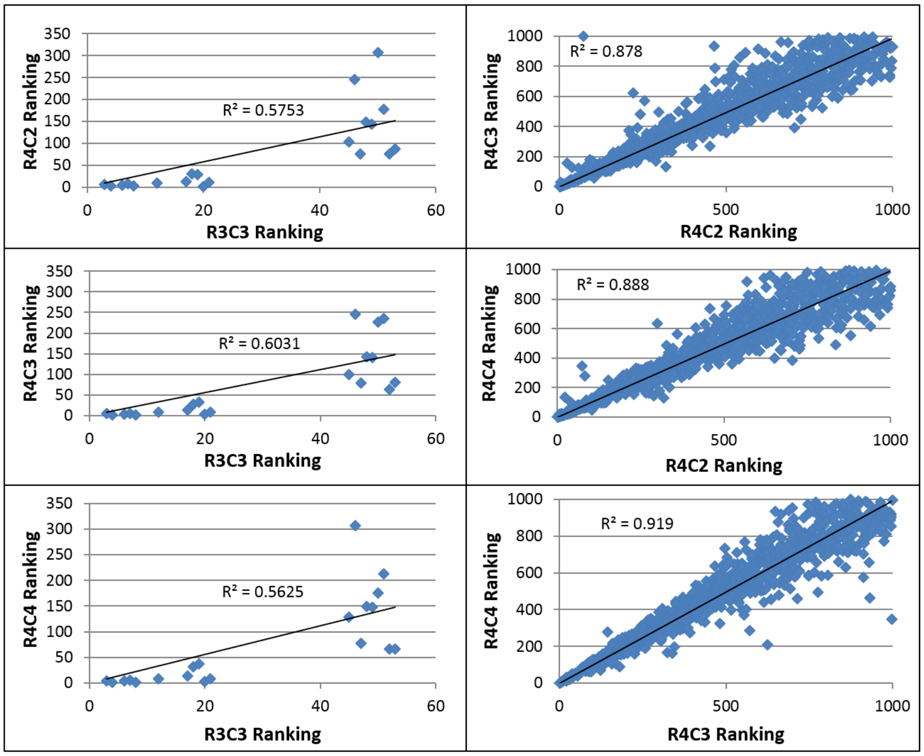

Supplementary Figure S1: Analysis of TIP60-Chromo enriched aptamer clusters through selection rounds and cycles. (a) Median multiplicity of top 1,000 TIP60-Chromo aptamer clusters in a given round-cycle and their median multiplicity in subsequent round-cycles of selection. (b) Number of common aptamer clusters in top 1,000 clusters between different round-cycles of selection for TIP60-Chromo. Common aptamer clusters from R2C1 to R3C2 with the later round-cycles are mostly due to sequence artifacts that pass through our sequence filter criteria. (c) Correlation plots for rankings of common aptamers in top 1,000 clusters at different selection cycles for TIP60-Chromo. R^2 values for each correlation are indicated. Multiplicities (reads per million) for 1st, 20th, and 1000th cluster in each library are as follows: R2C1 – 130.4, 1.3, 0.5; R2C2 – 184.5, 1.7, 0.6; R3C2 – 145.1, 2.5, 0.6; R3C3 – 697.4, 2.4, 1.2; R4C2 – 3,164.8, 610.4, 14.8; R4C3 – 12,038.3, 1,958.4, 43.6; and R4C4 – 7,923.3, 1,520.1, 35.8.

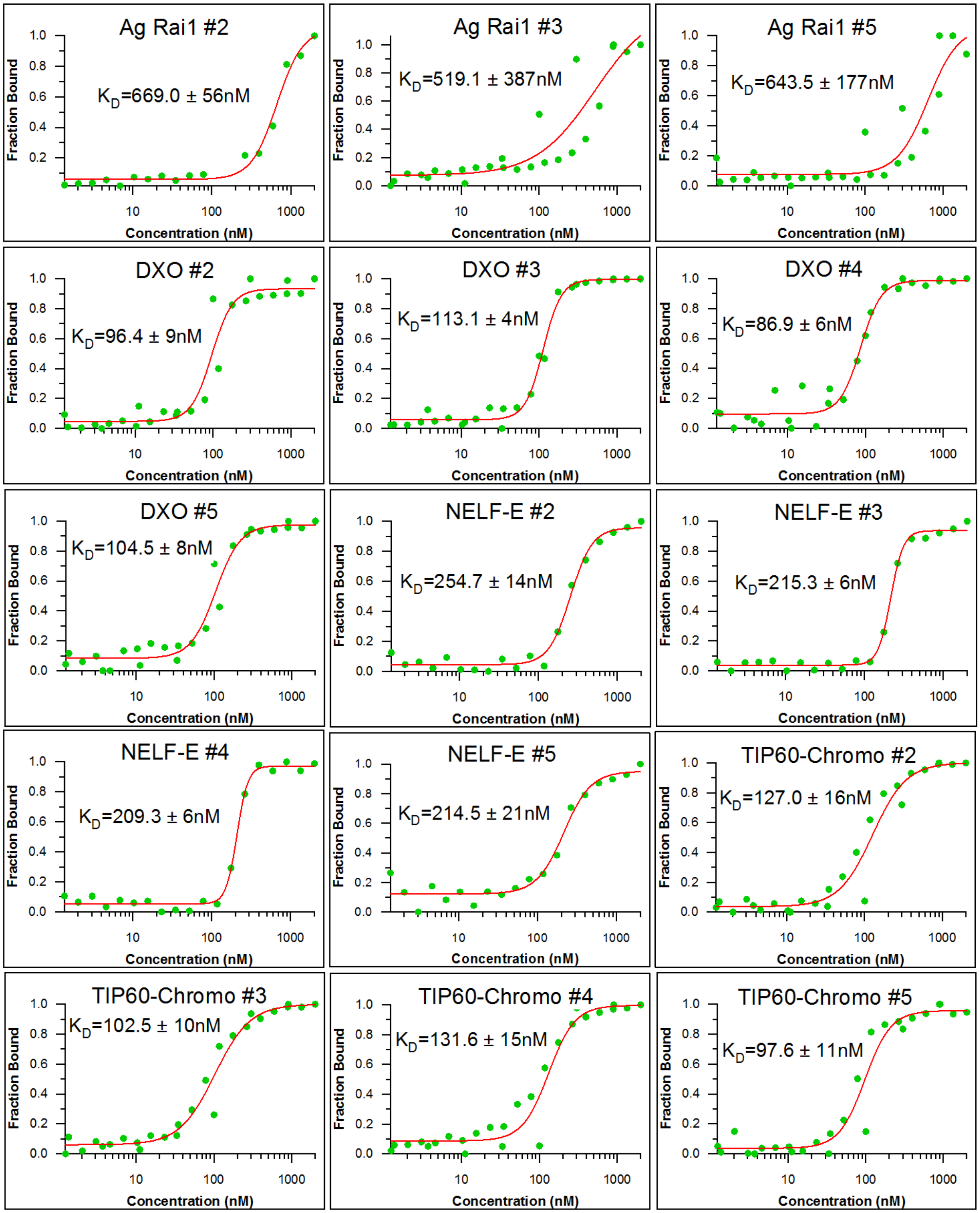

Ag Rai1 #2
K_D=669.0 ± 56nM
Ag Rai1 #3
K_D=519.1 ± 387nM
Ag Rai1 #5
K_D=643.5 ± 177nM
DXO #2
K_D=96.4 ± 9nM
DXO #3
K_D=113.1 ± 4nM
DXO #4
K_D=86.9 ± 6nM
DXO #5
K_D=104.5 ± 8nM
NELF-E #2
K_D=254.7 ± 14nM
NELF-E #3
K_D=215.3 ± 6nM
NELF-E #4
K_D=209.3 ± 6nM
NELF-E #5
K_D=214.5 ± 21nM
TIP60-Chromo #2
K_D=127.0 ± 16nM
TIP60-Chromo #3
K_D=102.5 ± 10nM
TIP60-Chromo #4
K_D=131.6 ± 15nM
TIP60-Chromo #5
K_D=97.6 ± 11nM
Fraction Bound
Concentration (nM)

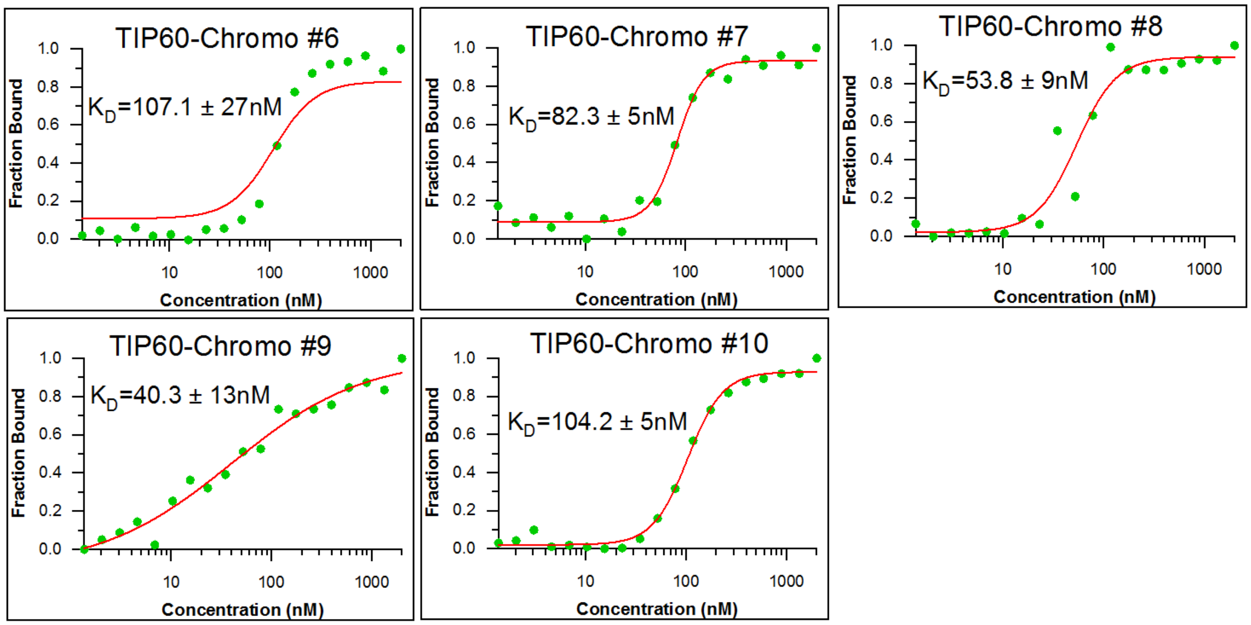

Supplementary Figure S2: Binding analysis of candidate aptamer sequences via EMSA. Results from EMSA experiments were quantified using ImageJ, and fitted to the Hill Equation using Igor Pro 5.04A.

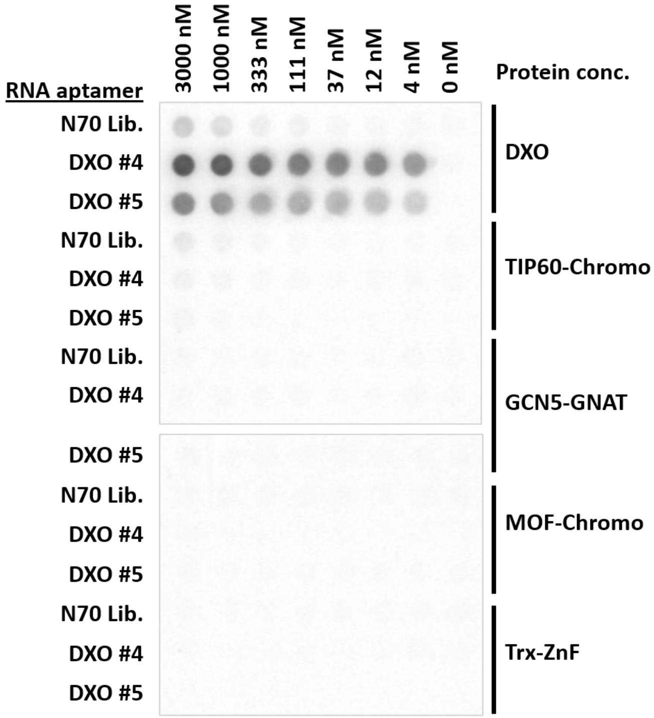

Supplementary Figure S3. Specificity of DXO aptamers. The specificity of DXO aptamers was tested by nitrocellulose filter binding assay. Radiolabeled RNA aptamers, DXO #4 and #5, and the N70 library (negative control) were incubated with varying concentrations (3000-4 nM) of DXO, TIP60-Chromo, GCN5-GNAT, MOF-Chromo, or Trx-ZnFinger proteins. Protein bound aptamers were captured by a nitrocellulose filter and detected by phosphorimaging. DXO #4 and #5 aptamers showed very weak binding, indistinguishable from N70 library control, against all other target proteins tested.

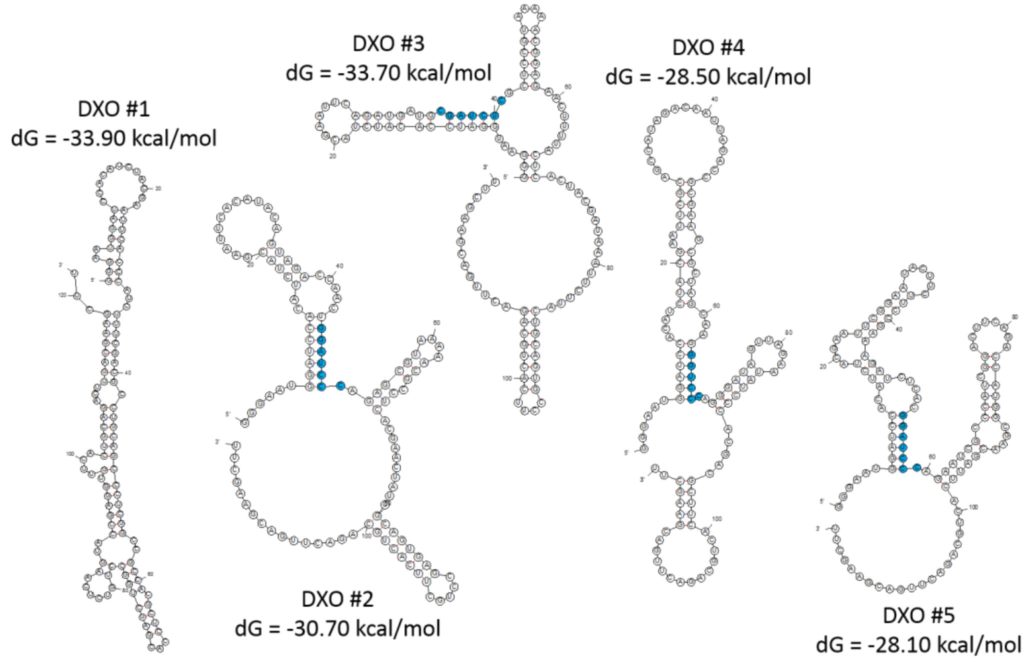

Supplementary Figure S4: Predicted secondary structures of DXO aptamers. MEME identified motif (DXO motif 1), which base-pairs with the BamHI site in the forward constant region of the N70 RNA library, is colored cyan. The predicted secondary structures and the deltaG values were obtained from mFold Web Server (http://unafold.rna.albany.edu/?q=mfold/RNA-Folding-Form).

a

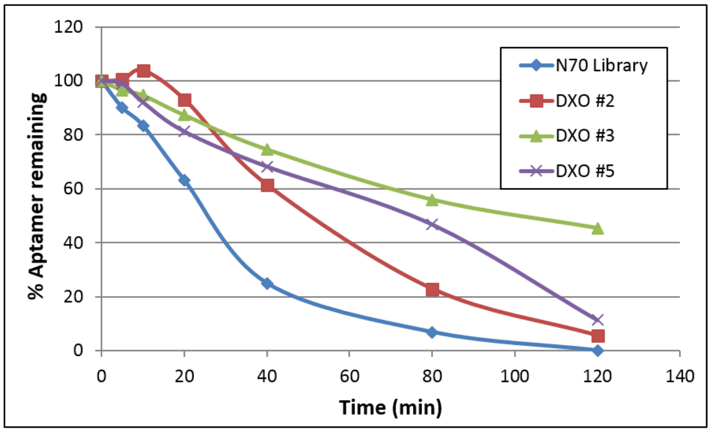

b

	$T_{1/2}$ (min)
**N70 Library**	19.8
**DXO #2**	28.9
**DXO #3**	43.3
**DXO #5**	69.3

Supplementary Figure S5: Stability of DXO aptamers against DXO exoribonuclease activity. (a) Each radiolabeled RNA aptamer was incubated with 1 μM DXO protein and samples collected at indicated time points were analyzed by running on an 8% denaturing PAGE gel. The gel image was analyzed, and a DXO exoribonuclease assay time course was plotted. (b) Aptamer half-lifes ($T_{1/2}$ min) are estimated from exponential decay function fit to measured % full-length aptamer remaining. Images were analyzed by ImageJ and data analysis was done in Microsoft Excel.

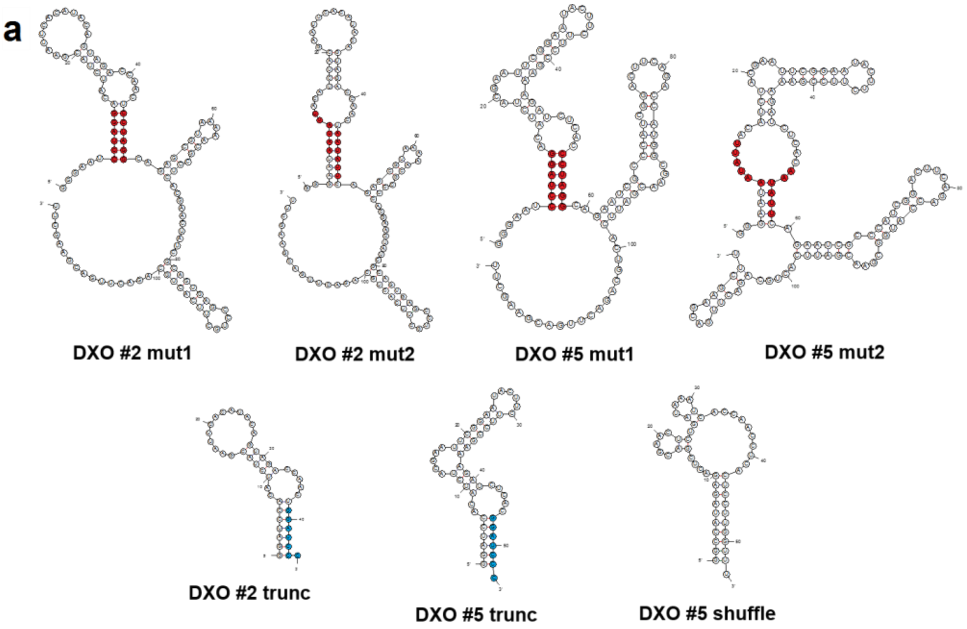

b

Aptamer	DXO #2	DXO #2 mut1	DXO #2 mut2	DXO #2 trunc	DXO #5	DXO #5 mut1	DXO #5 mut2	DXO #5 trunc	DXO #5 shuffle
$dG_{complete}$	-30.70 kcal/mol	-30.30 kcal/mol	-23.30 kcal/mol	-13.70 kcal/mol	-28.10 kcal/mol	-27.40 kcal/mol	-21.80 kcal/mol	-16.70 kcal/mol	-6.00 kcal/mol
$dG_{stem\#1}$	-8.40 kcal/mol	-8.30 kcal/mol	-0.20 kcal/mol	-8.40 kcal/mol	-7.60 kcal/mol	-7.20 kcal/mol	1.00 kcal/mol	-7.60 kcal/mol	-5.50 kcal/mol

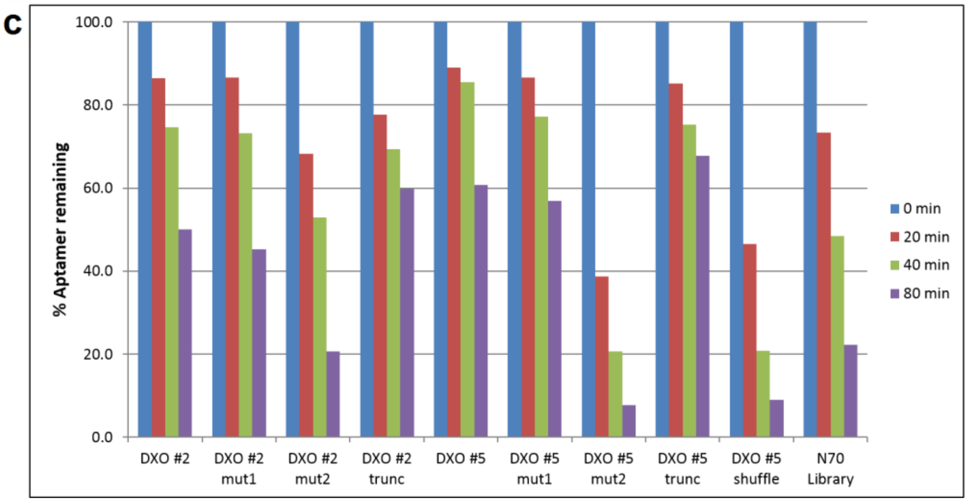

Supplementary Figure S6: Mutant and truncated DXO aptamers. (a) Predicted secondary structure of mutant and truncated DXO aptamers. MEME identified motif (DXO motif 1) is colored cyan and mutations to motif and the corresponding mutations in the forward constant region are colored red. In mut1, the original motif, GGATCCC, was changed to CCTAGGC and the BamHI site, GGATCC, in the forward constant region was changed to CCTAGG. In mut2, the original motif, GGATCCC, was changed to AATATTC and the BamHI site, GGATCC, in the forward constant region was changed to AATATT. Truncations of DXO #2 and #5 were made to retain the DXO motif 1 (cyan) and the first stem-loop structure for each aptamer. A sequence-shuffled version of DXO #5 trunc was used as a control. Secondary structures were predicted and the deltaG values were calculated by mFold Web Server (http://unafold.rna.albany.edu/?q=mfold/RNA-Folding-Form). (b) Stability (deltaG values) of complete predicted structure and the first stem of original full-length, mutant, and truncated versions of DXO aptamers. (c) Stability of original, mutant and truncated DXO aptamers against DXO exoribonuclease activity.

a

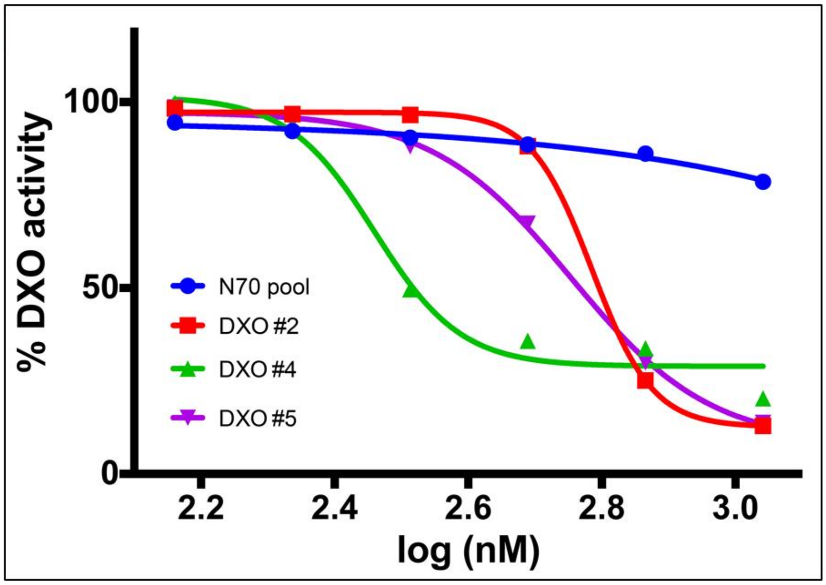

b

	IC50 (nM)
**N70 apt. pool**	>> 1100
**DXO #2**	609.5 ± 5.3
**DXO #3**	288.4 ± 29.8
**DXO #5**	567.5 ± 17.2

c

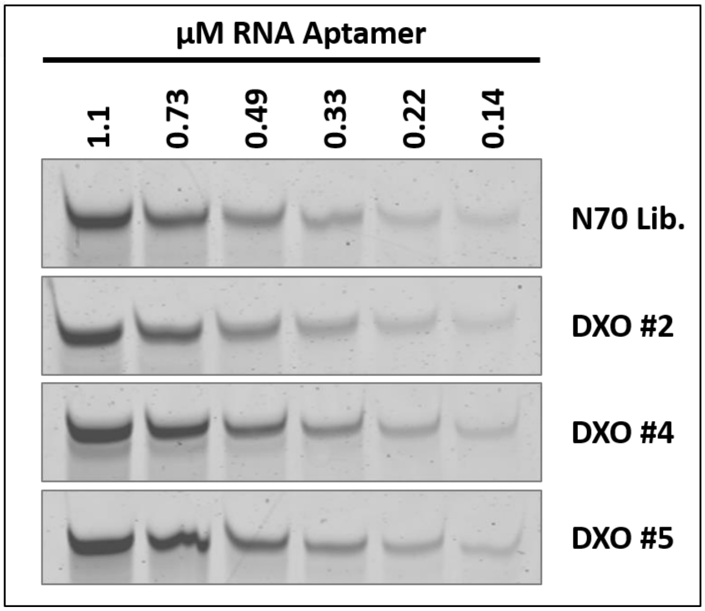

Supplementary Figure S7: Inhibition of DXO exoribonuclease activity by RNA Aptamers. (a) Quantification of DXO exoribonuclease activity against 3'-Cy5 labeled 30-nt RNA substrate in the presence of indicated concentrations of N70 aptamer pool, or DXO #2, #4, or #5 RNA aptamers from gels images shown in Fig. 4d. Data was fitted to a 4-parameter dose-response curve using GraghPad Prism6 software, and the estimated IC50 values are listed (b). Errors represent the standard error. (c) Verification of equal loading of RNA aptamers, which remained largely intact even after two hours of incubation with DXO enzyme, in DXO exoribonuclease inhibition assays.

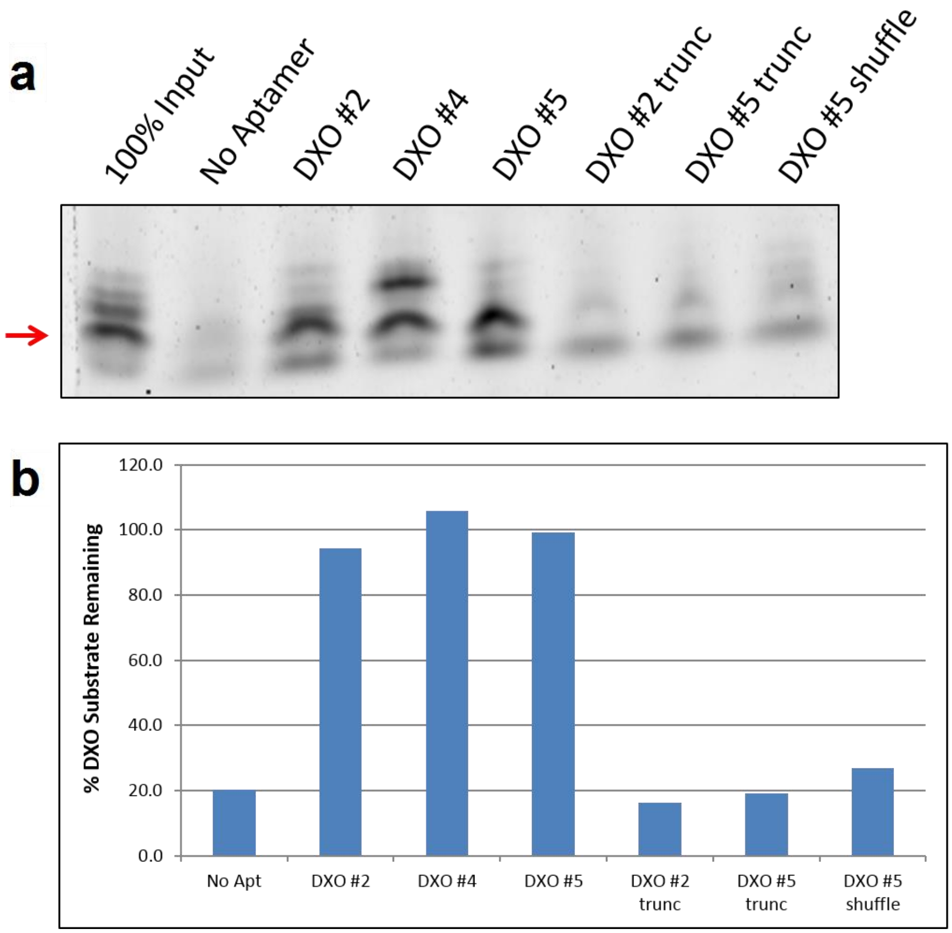

Supplementary Figure S8: Inhibition of DXO exoribonuclease activity by full-length or truncated DXO RNA aptamers. A) 3'-Cy5 labeled 30 nt RNA substrate was incubated with 1uM DXO exoribonuclease either in the absence of any aptamer (No Aptamer) or in the presence of 0.8μM full-length or truncated DXO RNA aptamers. After 2 hours of incubation at 37°C, exoribonuclease reactions were separated by 10% denaturing PAGE, and the remaining intact RNA substrate, indicated by →, was visualized by Cy5 fluorescence scan. B) Percentage of full-length DXO substrate remaining under each condition as quantified by ImageJ.
